# Publisher Correction: Seeded X-ray free-electron laser generating radiation with laser statistical properties

**DOI:** 10.1038/s41467-019-13249-4

**Published:** 2019-11-13

**Authors:** Oleg Yu. Gorobtsov, Giuseppe Mercurio, Flavio Capotondi, Petr Skopintsev, Sergey Lazarev, Ivan A. Zaluzhnyy, Miltcho B. Danailov, Martina Dell’Angela, Michele Manfredda, Emanuele Pedersoli, Luca Giannessi, Maya Kiskinova, Kevin C. Prince, Wilfried Wurth, Ivan A. Vartanyants

**Affiliations:** 1 Deutsches Elektronen-Synchrotron DESY, Notkestrasse 85, D-22607 Hamburg, Germany; 2 Department of Physics, University of Hamburg and Center for Free Electron Laser Science, Luruper Chausse 149, D-22761 Hamburg, Germany; 3 Elettra-Sincrotrone Trieste, 34149 Basovizza (Trieste), Italy; 4 National Research Tomsk Polytechnic University (TPU), pr. Lenina 30, 634050 Tomsk, Russia; 5 National Research Nuclear University MEPhI (Moscow Engineering Physics Institute), Kashirskoe shosse 31, 115409 Moscow, Russia; 6 CNR- IOM Istituto Officina dei Materiali, 34149 Trieste, Italy; 7 ENEA C.R. Frascati, Via E. Fermi 45, 00044 Frascati, Rome, Italy; 8 Molecular Model Discovery Laboratory, Department of Chemistry and Biotechnology, School of Science Swinburne University of Technology, Melbourne, VIC 3122 Australia; 9 Present Address: Department of Materials Science and Engineering, Cornell University, Ithaca, NY 14850 USA; 10 Present Address: European XFEL GmbH, Holzkoppel 4, D-22869 Schenefeld, Germany; 11 Present Address: Laboratory for Biomolecular Research, Division of Biology and Chemistry, Paul Scherrer Institute, PSI Aarebrucke, 5232 Villigen, Switzerland; 12 Present Address: Department of Physics, University of California San Diego, La Jolla, CA 92093 USA

**Keywords:** Free-electron lasers, Optical spectroscopy

Correction to: *Nature Communications* 10.1038/s41467-018-06743-8, published online 29 October 2018.

In the original version of the Article, the horizontal axis labels of Figure 4d incorrectly read ‘0, 05, 100’ instead of the correct ‘0, 50, 100’. This has been corrected in both the PDF and the HTML versions of the Article.

