# Supplementary material for: Seeded X-ray free-electron laser generating radiation with laser statistical properties
Source: Nat Commun. 2018 Oct 29;9:4498. doi: 10.1038/s41467-018-06743-8 (PMC6206026; doi:10.1038/s41467-018-06743-8)
Supplement: Supplementary file 1 — Supplementary Information [file 41467_2018_6743_MOESM1_ESM.pdf]

# Supplementary information for
# "Seeded x-ray free-electron laser generating radiation with laser statistical properties"

O. Yu. Gorobtsov, G. Mercurio, F. Capotondi, P. Skopintsev, S. Lazarev,
I.A. Zaluzhnyy, M. Danailov, M. Dell`Angela, M. Manfredda, E. Pedersoli, L. Giannessi, M. Kiskinova, K. C. Prince, W. Wurth, and I. A. Vartanyants

## Supplementary Note 1: Additional experimental results

Projections of the averaged intensity distribution (black lines) in the vertical and horizontal directions for the seeded and SASE modes of operation are shown in supplementary figure 1. It can be well seen from these figures that intensity distribution is significantly narrower in the seeded regime. Intensities of individual pulses (blue lines) are similar in shape to the averaged intensity that suggests that in the transverse direction a single mode is predominant.

Cross sections of the intensity correlation functions in the vertical and horizontal directions along the two diagonals (red and white) in supplementary figure 2 (a, d) of the main text for the seeded and SASE modes are presented in supplementary figures 2 and 3. The values below one (anticorrelation) in $g^{(2)}$-function (see supplementary figure 3) may appear due to positional jitter [1].

Intensity correlation functions in frequency domain measured at the spectrometer are presented in supplementary figure 4 and display very pronounced features typical for the jitter-affected intensity distribution [1] in the seeded regime (see supplementary figure 4 (a)). It suggests that significant jitter of the carrier wavelength was present during our measurements.

## Supplementary Note 2: Sorting of spectral modes

In order to analyze the spectral dependence of the $g^{(2)}$ function in seeded mode of operation, we implemented a sorting procedure. Spectra of individual pulses were fitted with multiple Gaussian distributions and sorted versus the relative percentage of energy in the most intense mode using the following procedure. Pulses containing multiple longitudinal modes generally contain several peaks in the spectral profile. In order to estimate the number and contribution of each mode, the spectral distribution of each pulse was fitted with several Gaussian distributions (multiple peak Gaussian distribution). The spectral profile of each

pulse was first fitted with one Gaussian function, then with two, and so on. The number of distributions was not increased further if one of the two conditions was fulfilled. First, when the unadjusted coefficient of determination $R^2$ reaches the value smaller than $10^{-4}$; or second, when the area under the most intense peak, that correspond to the power of the main mode, is decreased by more than 10% of the total area under the spectrum. Last criterion avoids overfitting of the data. Additionally, the maximum width (FWHM) of each Gaussian spectral distribution has an upper limit of 0.2 $fs^{-1}$ in order to avoid appearance of peaks wider than the whole pulse spectrum. By that we selected $10^3$ pulses with the largest contribution of the main mode and $10^3$ pulses with the smallest contribution.

## Supplementary Note 3: Analysis of intensity dispersion as a function of the bandwidth

We analysed dispersion of the total intensity $I_{tot}$ by using the two-dimensional intensity distribution on the spectrometer detector as a function of the radiation bandwidth for both regimes of operation. Typical images obtained at the spectrometer in the seeded and SASE modes of operation are shown in supplementary figure 5. By choosing different bandwidths around the carrier frequency and integrating intensity in the considered region, the value of the total intensity in the selected bandwidth for each pulse is determined. The relative dispersion is than calculated as

$$\zeta_{tot} = \frac{\langle (I - \langle I \rangle)^2 \rangle}{\langle I \rangle^2}. \qquad (1)$$

## Supplementary Note 4: FERMI operation

FERMI FEL-2 employs a double cascade of high gain harmonic generation (HGHG). The HGHG scheme [2,3] consists in preparing the electron beam phase space in a first undulator, called modulator, where the interaction with an external laser, the seed, induces a controlled, periodic modulation, in the beam energy distribution. The beam then traverses a dispersive section, which converts the energy modulation into a density modulation. The higher order harmonic components of this modulation retain the phase and amplitude properties of the seed. The density modulated beam is then injected into an FEL amplifier, resonant to the desired higher order harmonic, where the startup of the amplification process is enhanced by the presence of the modulation. The modulation depth may be tuned by varying the seed intensity or the dispersion, in order to reach saturation and efficient energy

extraction at the end of the amplifier. This HGHG scheme is implemented in FERMI FEL-1 to generate fully coherent radiation pulses in the VUV spectral range, from 100 nm to 20 nm. The amplitude of the energy modulation necessary to initiate the HGHG process grows with the order of the harmonic conversion. The induced energy dispersion has a detrimental effect on the amplification in the final radiator at higher harmonic orders. For this reason in FERMI FEL-2 the harmonic multiplication process is repeated twice. In a first HGHG stage an intermediate harmonic is produced, that is then used as a seed in a second HGHG stage. This double conversion is achieved with the fresh bunch injection technique [2,3] where a delay line slows down the electron bunch to accommodate the seed from the first stage on a fresh portion of the electron current, allowing the second harmonic conversion on electrons with low energy spread. FEL-2 is designed and operates in the spectral range from 20 nm to 4 nm, at the upper edge of the water window. This experiment was done with the seed initiating the cascade produced by a Ti:Sa oscillator, amplified in a regenerative amplifier and converted to the third harmonic at 261.5 nm. The first stage of FEL-2 was operated at the $6^{th}$ harmonic of the seed while the second stage was operated at the $4^{th}$ harmonic of the first stage, for a total harmonic conversion factor of 24, corresponding to 10.9 nm of final output wavelength. A seed energy of 15 μJ was sufficient to induce a coherent modulation in the electron current at the entrance of the amplifier three orders of magnitude larger than the shot noise background. This estimate suggests that the SASE background power was $10^6$ times lower than the seeded FEL power. In terms of energy we have to consider that the pulse duration in seeded mode is expected to be of the order of 40-50 fs [4] in the operating conditions of the experiment, while in SASE mode most of the beam, about 1 ps long, may contribute to emission. We would therefore expect to have an energy contrast between the seeded coherent signal and the SASE Gaussian noise of approximately $10^4$ - $10^5$.

This estimate does not include the effects of macroscopic or microscopic modulations of the beam current that could enhance the SASE emission or directly induce modulations of chaotic nature in the emitted radiation. The main player in this respect is the microbunching instability amplifying existing modulations in the beam that are then mixed with the energy modulation induced by the seed and may determine an observable effect on the FEL spectrum [5], as the formation of SASE like structures. The FEL operating conditions were set in order to minimize these effects. However, a diagnostic based on HBT interferometry is very promising in distinguishing the presence of microbunching instability and may contribute to the mitigation of microbunching instability during the FEL tuning process.

**Supplementary references**

[1] Gorobtsov, O. Y. *et al.* Statistical properties of a free-electron laser revealed by Hanbury Brown-Twiss interferometry. Phys. Rev. A **95**, 023843 (2017).

[2] Yu, L. H. Generation of intense UV radiation by subharmonically seeded single-pass free-electron lasers. Phys. Rev. A **44**, 5178 (1991).

[3] I. Ben-Zvi, K.M. Yang & L.H. Yu, Design of a harmonic generation FEL experiment at BNL. Nucl. Instrum & Meth. A **318**, 726 (1992).

[4] Finetti, P., et al., Pulse Duration of Seeded Free-Electron Lasers, Phys. Rev. X **7**, 021043 (2017).

[5] Roussel, E., Ferrari, E., Allaria, E., Penco, G., Di Mitri, S., Veronese, M., Danailov, M., Gauthier, D., & Giannessi, L., Multicolor High-Gain Free-Electron Laser Driven by Seeded Microbunching Instability. Phys. Rev. Lett. **115**, 214801 (2015).

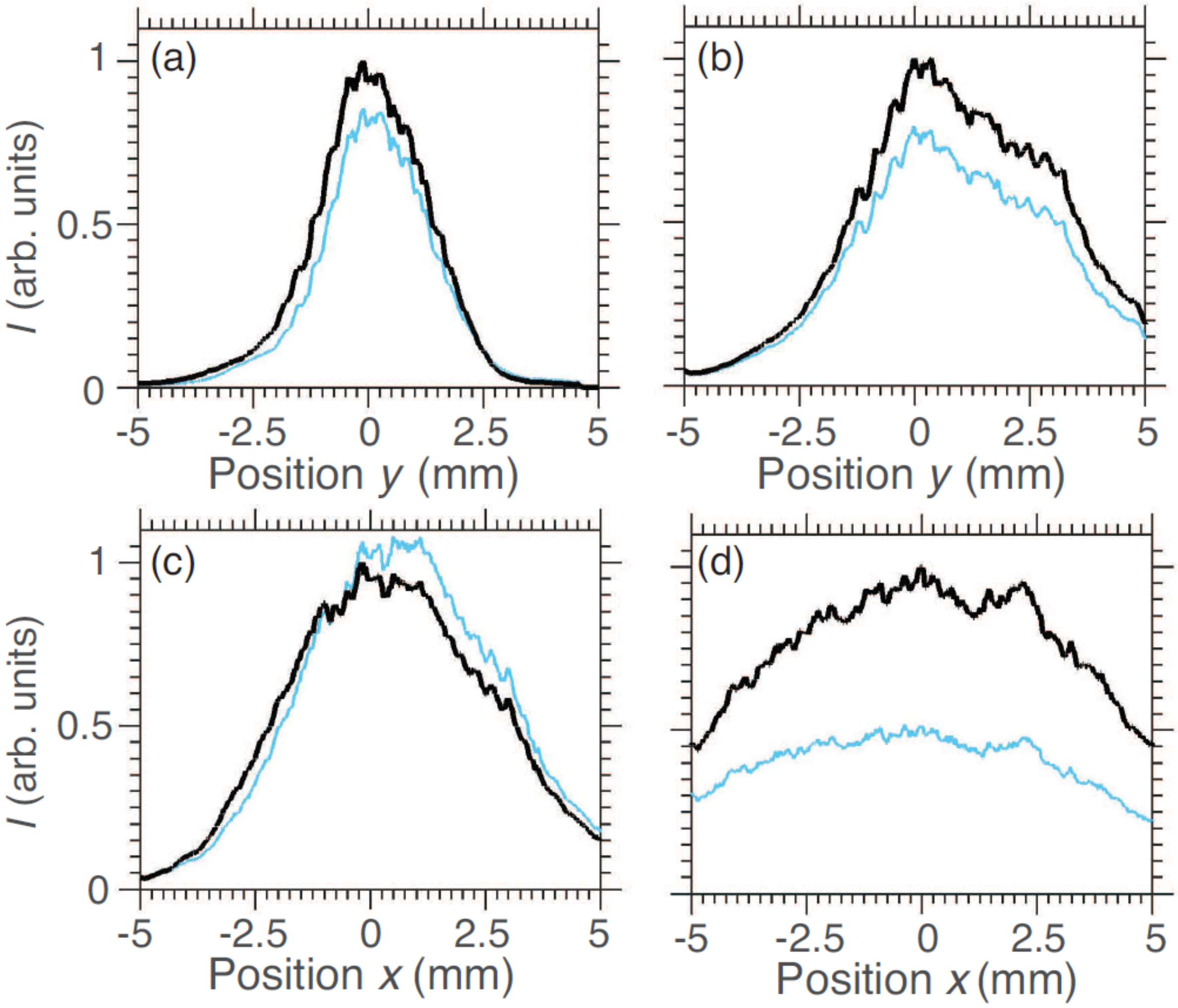


**Supplementary Figure 1:** Projection of intensity in different directions and modes of operation. Projection of the averaged intensity distribution (black lines) in the vertical (a,b) and horizontal (c,d) directions for the seeded (a,c) and SASE (b,d) modes of operation. Blue lines are individual pulses.

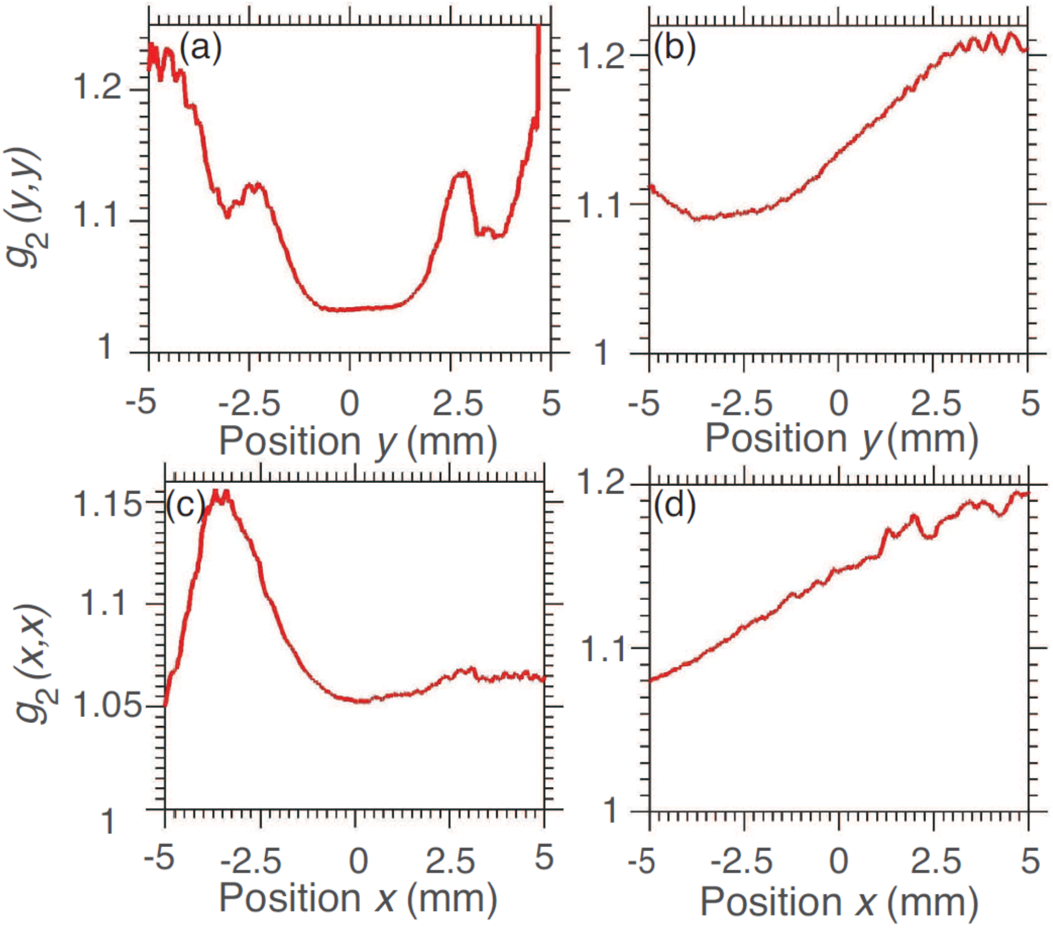


**Supplementary Figure 2:** Cross sections of the intensity correlation function. Cross sections of the intensity correlation function along the diagonal (red line) in Fig. 2 of the main text in the vertical direction (a, b) and in supplementary figure Fig. 2 in the horizontal direction (c, d) in the seeded (a, c) and SASE (b, d) regimes of operation.

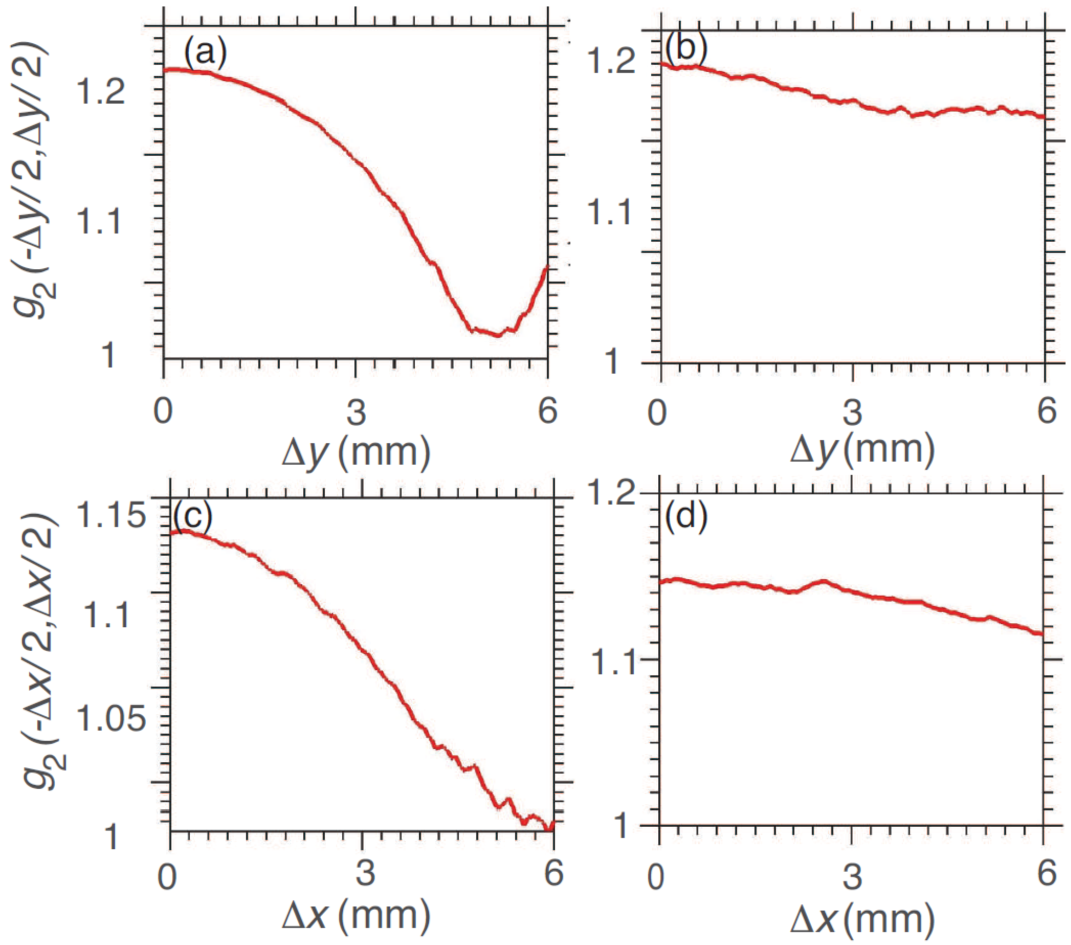


**Supplementary Figure 3:** Cross sections of the intensity correlation function. Cross sections of the intensity correlation function along the diagonal (white line) in Fig. 2 of the main text in the vertical direction (a, b) and in supplementary figure 2 in the horizontal direction (c, d) in the seeded (a, c) and SASE (b, d) regimes of operation.

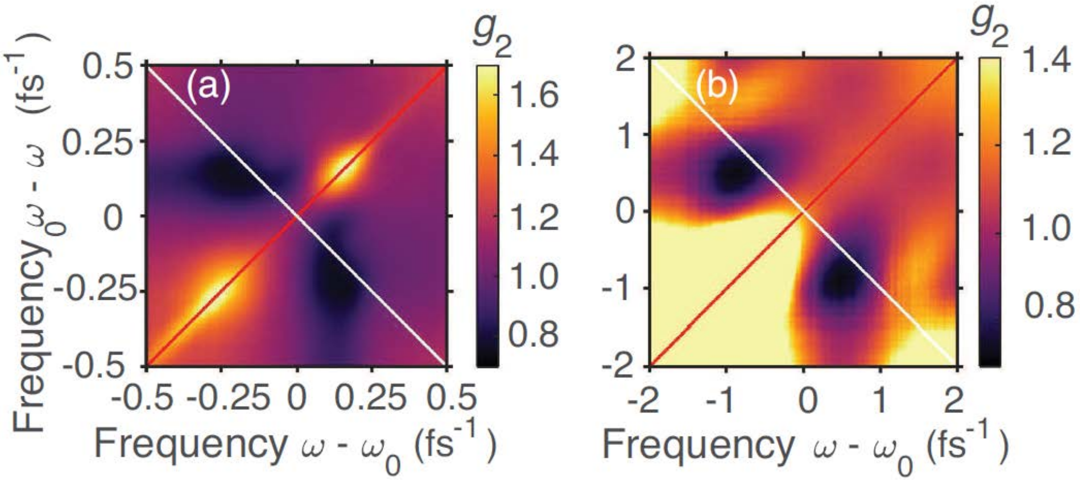


**Supplementary Figure 4:** Intensity correlation functions in frequency domain in different modes of operation. Intensity correlation functions in frequency domain measured at the spectrometer for seeded (a) and SASE (b) regimes of operation.

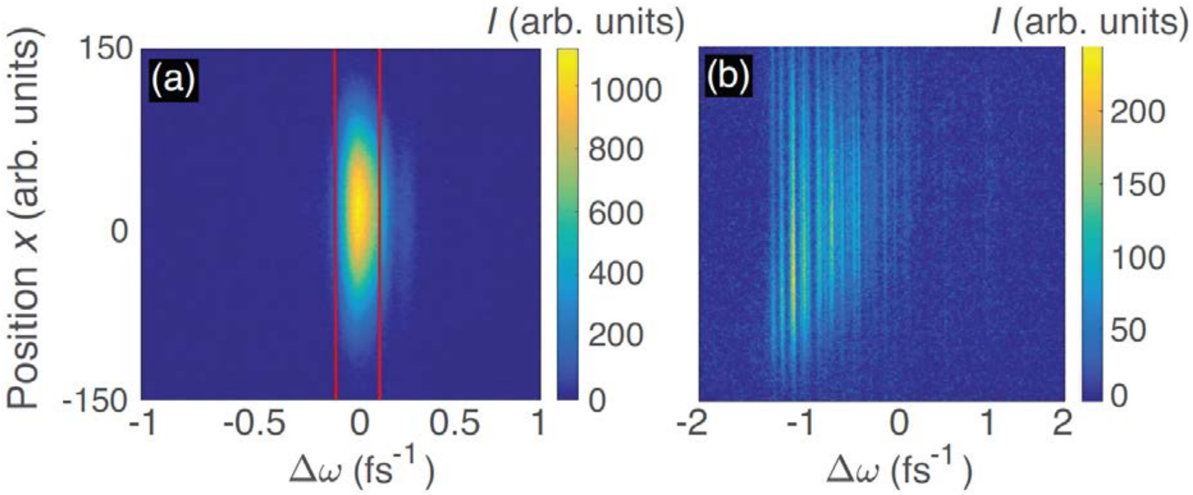


**Supplementary Figure 5:** Spectrometer detector images in different modes of operation. Typical images on the spectrometer detector in seeded (a) and SASE (b) modes of operation. Red lines show an example of a chosen bandwidth for analysis. Note twice wider spectral range in SASE mode.
